# Development of a Non-Invasive Machine-Learned Point-of-Care Rule-Out Test for Coronary Artery Disease

**DOI:** 10.3390/diagnostics14070719

**Published:** 2024-03-28

**Authors:** Timothy Burton, Farhad Fathieh, Navid Nemati, Horace R. Gillins, Ian P. Shadforth, Shyam Ramchandani, Charles R. Bridges

**Affiliations:** 1 Analytics for Life, Toronto, ON M5X 1C9, Canada; tim@analytics4life.com (T.B.); farhad.fathieh@analytics4life.com (F.F.); navid.nemati@analytics4life.com (N.N.); 2 Analytics for Life, Bethesda, MD 20814, USA; horace.gillins@analytics4life.com (H.R.G.); ian.shadforth@analytics4life.com (I.P.S.);

**Keywords:** front line, digital health, artificial intelligence, diagnostic

## Abstract

The current standard of care for coronary artery disease (CAD) requires an intake of radioactive or contrast enhancement dyes, radiation exposure, and stress and may take days to weeks for referral to gold-standard cardiac catheterization. The CAD diagnostic pathway would greatly benefit from a test to assess for CAD that enables the physician to rule it out at the point of care, thereby enabling the exploration of other diagnoses more rapidly. We sought to develop a test using machine learning to assess for CAD with a rule-out profile, using an easy-to-acquire signal (without stress/radiation) at the point of care. Given the historic disparate outcomes between sexes and urban/rural geographies in cardiology, we targeted equal performance across sexes in a geographically accessible test. Noninvasive photoplethysmogram and orthogonal voltage gradient signals were simultaneously acquired in a representative clinical population of subjects before invasive catheterization for those with CAD (gold-standard for the confirmation of CAD) and coronary computed tomographic angiography for those without CAD (excellent negative predictive value). Features were measured from the signal and used in machine learning to predict CAD status. The machine-learned algorithm achieved a sensitivity of 90% and specificity of 59%. The rule-out profile was maintained across both sexes, as well as all other relevant subgroups. A test to assess for CAD using machine learning on a noninvasive signal has been successfully developed, showing high performance and rule-out ability. Confirmation of the performance on a large clinical, blinded, enrollment-gated dataset is required before implementation of the test in clinical practice.

## 1. Introduction

Decreases in costs for data storage and computation have enhanced the applications of machine learning in cardiovascular medicine. Signals such as ECG and echocardiogram are ubiquitous, and, with relative ease, can be paired with clinical outcomes [1,2]. Further, machine learning algorithms can predict labels from data with high efficacy, for instance, ejection fraction estimation with echocardiogram [2] and arrythmia detection using ECG [3]. However, there is a relative dearth of successful applications of machine learning to more complex use cases, such as those with low prevalence, and those requiring invasive gold standard adjudication. A common error that contributes to this absence is the use of data that are mismatched with the intended use population, such as including healthy control subjects in the non-diseased cohort (erroneously augmenting test specificity [4]).

In recent years, deep learning has emerged as a focal point within the field of cardiology, particularly in contexts in which cardiac imaging data are accessible. Despite its remarkable capabilities in disease detection and the identification of cardiac events from imaging data, the application of deep learning in electrocardiography (ECG) remains mainly constrained to scenarios in which clear manifestations of disease or cardiac events are evident on ECG traces. These include tasks such as arrhythmia detection, atrial fibrillation (AF) detection, classification of ST events, ECG wave localization, and detection and classification of myocardial infarction (MI) [5]. A pitfall that can exasperate the usage of inappropriate data is the need for large-scale datasets by deep learning algorithms (10s to 100s of thousands), therefore biasing the practitioner to use flawed sources to supply sufficient data due to the typical unavailability of appropriate data of such magnitude in clinical applications. Deep learning algorithms benefit the practitioner in that the features (measurable characteristics of the data) are learned from the raw data by the model itself, thereby eliminating the need for domain knowledge to engineer features. However, the resultant learned features can stymy interpretability, which is of particular importance in clinical applications. In contrast, a return to classical machine learning that accepts features as input, rather than raw data, may address the limitations of deep learning in complex medical applications by reducing data requirements by orders of magnitude. Furthermore, knowledge of how the features are calculated and, consequently, which physiological characteristics they represent can assist in demystifying the “black box” perception of machine-learned algorithms, which is a necessity identified by the American Medical Association [6]. However, such an approach requires domain knowledge in signal processing, mathematics, and medicine to generate meaningful features.

Based on both ESC [7] and ACC [8] guidelines, standard point-of-care testing is typically followed by a more complex and rigorous assessment, involving a new encounter at a different care center. Over 80% of the patients are evaluated with (single photon emission computed tomography (SPECT) and/or coronary computed tomographic angiography (CCTA)) that can expose the patient to significant radiation and/or stress (pharmaceutical or exercise-induced) [9]. Testing may take place over a period of days to weeks, and positive/inconclusive results may result in referral to gold-standard invasive catheterization and possible intervention (i.e., catheter-based coronary intervention or coronary artery bypass graft surgery) if indicated by the presence of obstructive CAD. The American College of Cardiology (ACC) has defined obstructive CAD as the presence of a functionally significant lesion (FFR < 0.80 or iFR < 0.89) or, absent a functional assessment, a lesion of ≥70% (or 50% in the left main artery); when this definition is met, intervention is often warranted [8].

CAD diagnosis is especially difficult for rural populations, who often experience challenges in accessing comprehensive cardiovascular care and diagnostics. For instance, only 39% of rural counties were found to have access to point-of-care ultrasound, in comparison to 89% of metropolitan counties [10]. The lack of comprehensive care translates into outcome disparities. In patients <65 years of age, mortality from CAD is consistently higher in rural vs. urban populations (~1.7-fold increase for women, and ~1.5-fold increase for men) [11]. Significant sex differences also exist in the diagnosis of CAD. Specifically, risk stratification for CAD is less effective for women, resulting in lower angiographic diagnostic yield, which may be an overcompensation for the historic underdiagnosis of heart disease in women [12]. Indeed, a large registry review of almost 400,000 patients undergoing invasive catheterization found that while women were referred at a similar rate as men (47% vs. 53%), only 27% of women were found to have obstructive disease (vs. 47% of men) [13].

The duration of the CAD diagnostic pathway has at its source the lack of an effective, geographically accessible test at the point of care that is performant for both sexes. Ideally, such a test would rule out CAD, with high sensitivity and high negative predictive value (NPV). When this rule-out test is negative, the physician can confidently explore other non-CAD explanations. When it is positive, referral to a cardiologist for further evaluation is often warranted. Balanced performance between the sexes is of critical importance for such a test, given the historic diagnostic shortcomings experienced by women. Therefore, we sought to develop a machine-learned model using feature engineering (based on an easy-to-acquire signal) and classical machine learning with a rule-out profile that performs equally on each sex using a clinically relevant population.

## 2. Materials and Methods

### 2.1. Clinical Studies and Population

The population that we intend to be appropriate for evaluation with the trained algorithm is symptomatic patients at suspicion for CAD. Therefore, we designed studies to enroll subjects both with gold-standard confirmation of the presence of CAD using invasive coronary angiography (ICA) with left heart catheterization, as well as the vast majority of subjects who receive a negative result (with high NPV) from CCTA.

The subjects used in the present work were drawn from the CADLAD (NCT02784197) and IDENTIFY (NCT03864081) studies, both approved by the Western Institutional Review Board. Informed consent was obtained from all subjects. CADLAD enrolled subjects prior to ICA. The CorVista Capture device [14] encompasses all the hardware and software necessary to simultaneously acquire photoplethysmogram (PPG) and orthogonal voltage gradient (OVG) signals from the subjects. Signals undergo a signal quality assessment and outlier detection [15], and upon successfully passing through the quality modules, features are extracted, which are then utilized for model development and the generation of predictions. The IDENTIFY study continues and extends CADLAD; IDENTIFY Group 2 is identical to CADLAD, while IDENTIFY Group 4 enrolled subjects prior to CCTA, representing all subjects who received diagnostic testing with a subsequent negative result. See inclusion/exclusion criteria in Supplementary Section S1. The other groups within IDENTIFY were not relevant to the present work.

The CADLAD and IDENTIFY Group 2 population are those referred for ICA (gold standard to confirm presence of CAD); this population represents about 10% of subjects who initially enter the CAD diagnostic workflow [16]. Approximately 62% of these subjects will be CAD− from ICA [13,16] (i.e., CAD diagnostic workflow false positive). The remaining 38% are CAD+ (CAD diagnostic workflow true positive) and are the source of CAD+ subjects for the present work, as defined by ACC guidelines [8]. Because 10% of subjects are referred for catheterization, CAD has a low prevalence of 3.8% in the overall symptomatic population referred for diagnostic testing. The remaining 90% are not referred for cardiac catheterization because of one or more negative test results in the diagnostic workflow, often SPECT or CCTA, the latter of which has an excellent NPV [17] (especially given the low disease prevalence). Therefore, the negatives from CCTA are the CAD− subjects in the present work, which are defined as a 0–2 on the Coronary Artery Disease Reporting and Data System (CADRADS) scale [18].

### 2.2. Overview of Development Process

Features were calculated from the OVG and PPG signals [14,15], and univariate feature selection was used to identify features with statistical power for CAD. Machine learning was then used to associate the selected features with the CAD status. After machine learning, cut points were selected and applied to the prediction (CAD score) for each sex and performance characterized. The resultant processing pipeline to generate the prediction, which comprised extracting the selected features, applying the machine-learned models, and applying the cut point to delineate test-negativity from test-positivity, is referred to as the CAD algorithm.

### 2.3. Data

The function of the datasets is shown in Table 1. Training and internal validation refers to iteratively training and generating naïve predictions within cross-validation for performance evaluation. CADLAD contributed N = 624 subjects in total, composed of N = 416 CAD+ and N = 208 CAD−. The data were segmented into N = 416 for feature selection, with all N = 208 CAD− and half of the CAD+, N = 208. All the N = 416 CAD+ were used for training and internal validation, including the N = 208 CAD+ subjects used for both purposes. IDENTIFY Groups 2 and 4 also contributed to training and internal validation, supplying N = 225 and N = 513, respectively. CADLAD and IDENTIFY Group 2, both representing CAD+ from the catheterization population, and IDENTIFY Group 4, representing CAD− from the CCTA population, defines the intended use dataset—i.e., the dataset that represents the population for which the device is designed to be used. The validation (in training and internal validation) is a performance estimate only, given that the gold standard methodology for validating a machine-learned algorithm is a large, blinded dataset that is assessed only once—assessing the algorithm in such a manner is planned future work.

### 2.4. Dimensionality Reduction by Feature Selection

A feature library of 3298 measurements on the OVG and PPG in isolation, and in combination, has been developed [14,15,19]. Given the ratio of features to subjects in training, the curse of dimensionality limits effective learning due to challenges such as sparsity of the feature space [20]. Therefore, each feature was assessed individually for statistical ability to identify CAD. Statistical testing was performed on an N = 416 subset of CADLAD (Table 1), which was composed of N = 208 CAD− and N = 208 CAD+ subjects. Care was taken to ensure that the sexes were treated evenly. In this case, each N = 208 was composed of 104 females and 104 males.

Three univariate statistical tests were used to assess each feature, including *t*-test, with a strict threshold of *p* < 0.01; area under the receiver operator characteristic curve (ROC-AUC), in which the 95% bootstrap lower confidence bound threshold was raised from the convention of ROC-AUC > 0.50 to ROC-AUC > 0.52, and inverse feature predictivity was also considered (i.e., ROC-AUC < 0.48); and mutual information (MI), an information theoretic measure which detects differences between distributions [21]. The 95% lower confidence bound bootstrap threshold was set at 1.2 (1.0 indicates no difference). These tests assess differing mechanisms of predictivity; *t*-test detects linear differences (well-suited to linear ML models), while both ROC-AUC and MI detect nonlinear differences (well-suited to nonlinear ML models).

### 2.5. Out-Of-Fold Prediction

**Figure 1:**
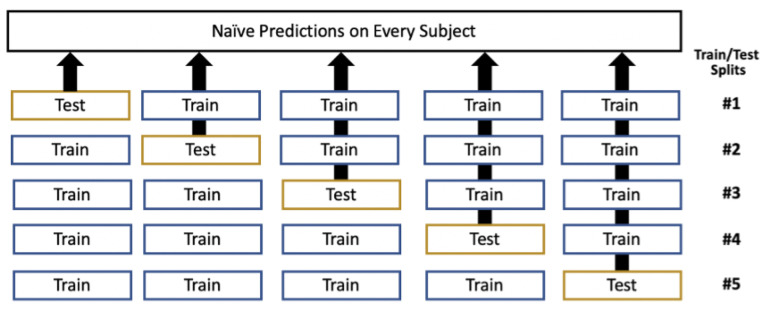
Process to generate out-of-fold predictions on each subject by extracting the naïve predictions from each test fold during cross-validation.

Cross-validation is the iterative segmentation of a dataset into different training and test sets, and here specifically, stratified (by CAD) 5-fold cross-validation [22]. Out-of-fold (OOF) prediction captures the naïve predictions from cross-validation. A single iteration of OOF through cross-validation generates five trained models (stored for later use) and a single naïve prediction for every subject, as shown in Figure 1. The result is averaged over 100 iterations to ensure variability in the fold generation. Over those iterations, OOF generates 500 models, which are applied to new data through averaging. OOF is a critical ML methodology given a relatively small dataset, enabling all the data to be used for training and testing, while generating a prediction for each subject for subsequent analysis (ROC curve plotting, ROC–AUC calculation, robust subgroup analysis). OOF has been previously utilized in a similar fashion [23]. The detriment of OOF is that any given model only has access to 80% of the data for training, which is an acceptable tradeoff.

### 2.6. Modeling

**Figure 2:**
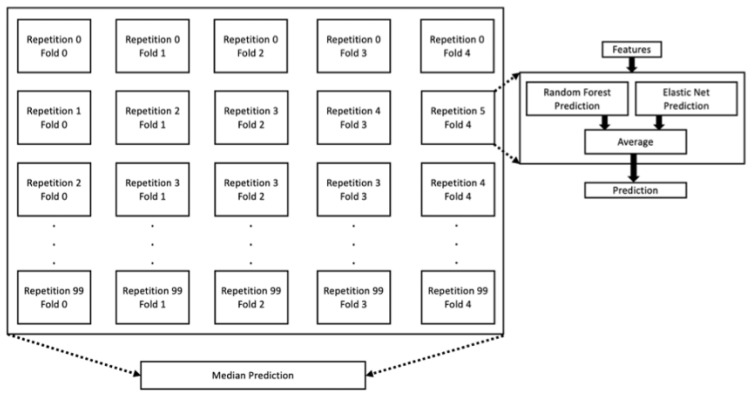
Process to generate a prediction on a new patient using the trained CAD algorithm; each individual model is a stacked ensemble of random forest and elastic net, in which the prediction from each is averaged. There are 500 of those models, defining a bagged ensemble (given that each was trained on different subsets of the overall dataset), which are combined using the median.

Two model types were selected for inclusion in the CAD algorithm, including elastic net (EN) and random forest (RF). EN is a linear model that regularizes weights (w) with $l_{1}$ (${\left\|w\right\|}_{1}=\sum_{i}w_{i}$) and $l_{2}$ (${\left\|w\right\|}_{2}^{2}=\sum_{i}w_{i}^{2}$) penalties [24]. EN is particularly effective when the number of features is large compared to the number of training subjects [25]. RF is an ensemble algorithm composed of underlying tree models [26]. Each tree optimizes the mean squared error loss function ($\frac{1}{n}\sum_{i=1}^{n}{\left\{y-\hat{y}\right\}}^{2})$ by selecting features upon which to split the dataset until a terminal node containing the prediction is reached. A large collection of trees is trained on differing subsets of the subjects, and the predictions from the trees are averaged. The usage of EN and RF allows both linear and nonlinear relationships, respectively, to be captured between features and CAD. The CAD algorithm is the average (i.e., stack) of EN and RF, with models trained across differing subsets of the overall dataset to define an overall bagged ensemble. Compression across the bagged ensemble is performed using median, with the overall CAD algorithm invocation process shown in Figure 2.

Equal treatment of both sexes was also applied in machine learning. The intended use dataset displayed an imbalance of females across the CAD− (65%) and CAD+ (27%, like the 34% rate in the large registry discussed previously) subgroups. Without mitigation, machine learning may leverage sex as a confounder for CAD. The first mitigation was sample weights to adjust for the relative frequencies of each sex/CAD subgroup. After training, the second mitigation was applied, which was the selection of sex-specific cut points delineating test positivity from negativity. Each cut point was selected at 90% sensitivity to ensure a consistent rule-out test profile for both sexes. Cut points were subtracted from the predictions to standardize at zero.

## 3. Results

### 3.1. Demographics and Disease in the Intended Use Dataset

As shown in Table 2, CAD+ subjects are older, with a higher frequency of males and more cardiovascular risk factors (hypertension, diabetes, and hyperlipidemia) but are absent BMI elevation. CAD severity is evenly distributed, with similar frequency of single- and multi-vessel disease, as well as CADRADS scores.

### 3.2. Feature Selection

A total of 290 features were selected. The most common scenario was *t*-test alone (68 features), followed by MI alone (64), then both *t*-test and AUC (52), with the remaining permutations accounting for the remaining 106.

### 3.3. Relationship between Elastic Net and Random Forest

**Figure 3:**
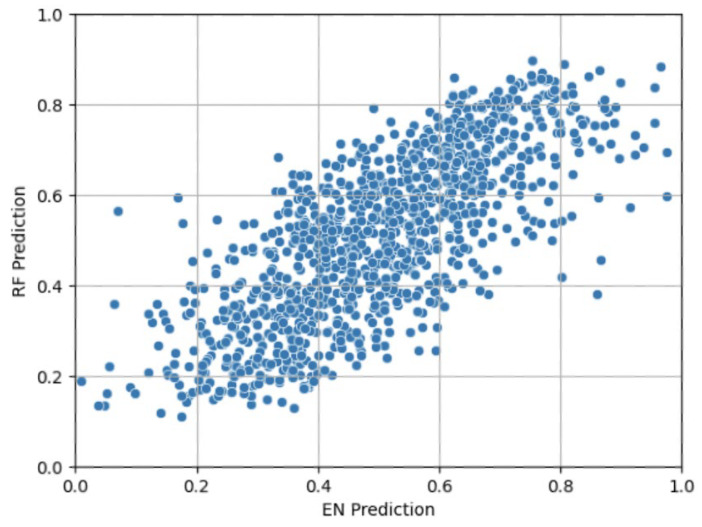
Scatterplot of elastic net (EN) prediction vs. random forest (RF) prediction, exhibiting only a modest Pearson correlation of 0.74.

The EN and RF OOF predictions have a moderate Pearson correlation of 0.74 (Figure 3). A high correlation is the baseline assumption, given that the models were trained on the same subjects and features. However, because EN is linear and RF is nonlinear, they had different usages of the features, thereby demonstrating the value of stacking.

### 3.4. Model Performance

**Figure 4:**
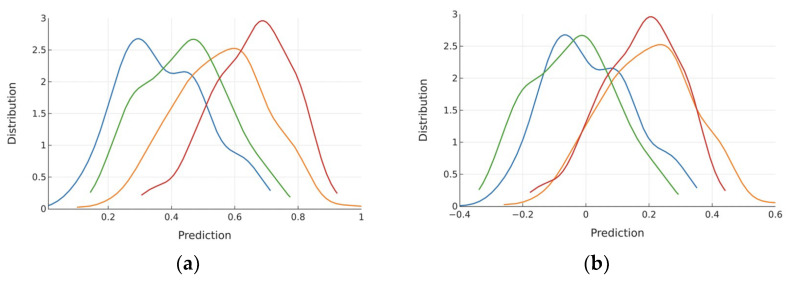
Predictions from female/male CAD+ (red/orange), female/male CAD− (green/blue) prior to (**a**) and after cut point subtraction (**b**).

The non-zeroed distribution of CAD Scores by sex/CAD subgroups are shown in Figure 4a. Male CAD− subjects are predicted more strongly negative than the female CAD− subjects (corresponding to the more substantial sample weight required to correct the proportion), and the opposite effect is seen in CAD+ subjects. To achieve 90% sensitivity in females, the female-specific cut point was 0.482, with a specificity of 61%. To achieve 90% sensitivity in males, the male-specific cut point was 0.361, with a specificity of 54%. After the cut point subtraction for each sex, the distributions shifted (Figure 4b), resulting in an overall sensitivity of 90% and specificity of 59% for both sexes.

**Figure 5:**
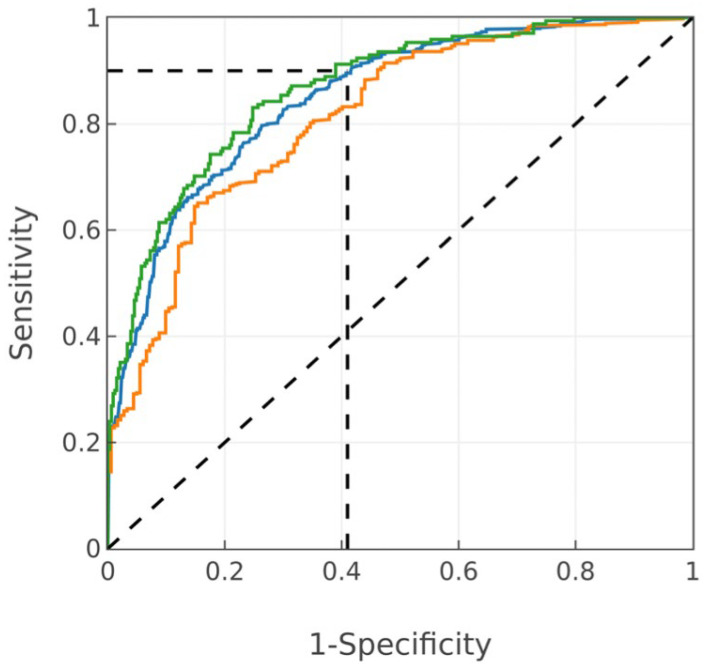
ROC curves using the CAD score (OOF post-zeroed ensemble predictions) for both sexes (blue, AUC = 0.85), males (orange, AUC = 0.81) and females (green, AUC = 0.87). Vertical and horizontal dotted lines intersect at a sensitivity and specificity on both sexes of 90% and 57%, respectively (corresponds to the cut point of zero).

The ROC curves for the male and female subgroups, as well as both combined (including cut point subtraction for each sex) is shown in Figure 5.

Recalling that N = 208 of the CAD+ subjects were used for both feature selection and ML (of N = 641, 32%), the sensitivity of this subgroup was 88%, confirming the absence of bias from multiple uses; the performance of this subgroup, as well as males, females, and both sexes together is shown in Table 3a. The CAD algorithm 2 × 2 performance is shown in Table 3b, with a resultant odds ratio of 12.12. Additional performance metrics for the model, i.e., Matthews correlation coefficient (MCC), area under the precision recall curve (AUC-PR), and F1-score, are provided in Supplementary Section S3.

### 3.5. Model Performance in the Presence of Confounders

The differences between the CAD+ and CAD− datasets shown in Table 2 represent possible confounders for the CAD algorithm performance, specifically with respect to those exhibiting statistical significance (*p* < 0.05), including sex, age (≥65), diabetes, hypertension, and hyperlipidemia. Therefore, it is critical to analyze performance by subgroups, which showed that the rule-out profile is maintained across each (Supplementary Section S4).

Further, Table 3b demonstrates that the CAD algorithm has an odds ratio of 12.12, but that is raw in the sense that it is not adjusted for any of these possible confounders. Therefore, a multivariate logistic regression was performed to generate an odds ratio for each of these factors with respect to CAD status, alongside the test-positive CAD score, as shown in Table 4 (sorted by descending odds ratio). The CAD score exhibited an adjusted odds ratio of 7.50 and remained the strongest predictor of CAD despite the demographic differences.

### 3.6. Feature Importance

**Figure 6:**
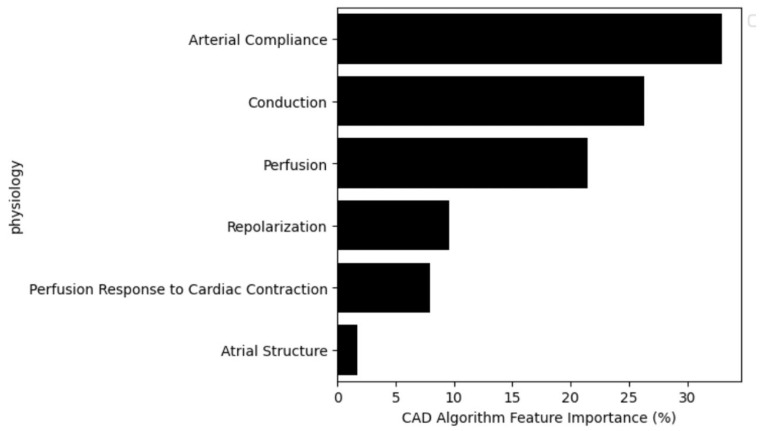
Analysis of the CAD algorithm feature importance by physiological category.

Lack of transparency can be a significant barrier to the adoption of AI systems, especially in healthcare domains in which understanding the reasoning behind decisions is essential. This study addresses this issue by employing explainable artificial intelligence’s (XAI) methods to compute feature importance [27]. Figure 6 illustrates the feature importance by physiological category. Further information is available in Supplementary Section S5. Arterial compliance is the most contributive, comprising features calculated using the PPG first or second derivative (i.e., velocity and acceleration plethysmograms), which embed characteristics of arterial compliance [28]. Conduction is the next most influential category, calculating characteristics of myocardial conduction pathway and variations in that pathway (Supplementary Section S5). Perfusion features capture PPG waveform morphology and the relationship between the infrared and red signals [29]. Repolarization features quantify myocardial recovery, including power distribution, heterogeneity, timing, morphology, and variation [30]. Perfusion response to cardiac contraction features characterize the OVG–PPG interplay, thereby embedding the perfusion response to cardiac pulsation [14]. Finally, atrial structure features capture atrial heterogeneity, including enlargement [31].

## 4. Discussion

A CAD algorithm was developed on a clinically relevant population; it was designed to perform equally on men and women with a rule-out profile. Initial performance using OOF predictions demonstrates that these design goals were met. The overall performance is a ROC–AUC of 0.85, with a sensitivity of 90% and specificity of 59%, resulting in a highly effective NPV of 99.32% at the CAD prevalence of 3.8%. Further, because sensitivity is high while specificity is maintained, the NPV continues to be effective at hypothetical higher prevalences, as follows: 98.12% at 10% prevalence, 95.87% at 20% prevalence, and 93.12% at 30% prevalence.

The performance is comparable to CCTA [9], yet CCTA is only available in a tertiary care setting. Our test, in contrast, requires no radiation exposure, no stress of any kind, nor contrast agents and can be performed in any setting, including rural (only requiring internet), with immediate results. Critically, the test addresses the disparity in healthcare access for rural vs. urban populations, given the portability and ease of application. Further, performance in females is similar, if not superior, to males. In contrast, current standard of care noninvasive testing for CAD performs significantly poorer on females [32,33].

The clinical dataset in the present work is substantial and accurately represents the intended use population, yet it presents challenges for deep learning approaches (requiring orders of magnitude more data). In contrast, to address the dataset size, we used engineered features and feature space dimensionality reduction, followed by classical machine learning using a stacked ensemble of EN and RF. The severe imbalance between CAD and sexes was successfully managed using sample weighting and sex-specific cut points.

A key advantage of classical machine learning, such as EN and RF, is ease of model interpretation, which, in contrast, is complex in deep learning. The feature importance analysis showed that the six most influential categories are arterial compliance, conduction, perfusion, repolarization, perfusion response to cardiac contraction, and atrial structure. Any feature alone is not definitively indicative of CAD. Further, none measure ischemia directly. However, each measures some aspect of cardiac/arterial function, and the machine learning models have found a complex relationship between them that can indicate the presence of CAD.

The first limitation is that performance reporting is based on OOF predictions, yet the gold-standard validation of a machine-learned model is a large, blinded dataset that is assessed only once—that testing is planned future work. A second limitation is the necessity for sex-specific cut points despite the use of sample weighting due to the shift observed in Figure 4a. Ideally, the sample weights would have immediately established the alignment in Figure 4b; because that was not observed, it is possible that there is a more efficient method to obtain sex alignment, which will be explored in future work. Note that this limitation exists largely due to the low number of CAD+ women in the dataset, which also prompted our decision to use OOF rather than the traditional approach of a single hold-out dataset for testing. Specifically, it was critical to expose the models to as many CAD+ women as possible to ensure that these patients would be detected in subsequent uses of the CAD algorithm.

## 5. Conclusions

The study outlined the development of a machine learning model for the detection of coronary artery disease (CAD) utilizing a noninvasive test, without the need for radioactive dyes, contrast enhancement, stress, or radiation exposure. The test aims to enable physicians to rule out CAD at the point of care, thereby facilitating the faster exploration of alternative diagnoses for symptomatic patients suspected of having CAD. The algorithm achieved a sensitivity of 90% and specificity of 59% with AUC–ROC 0.85, with consistent performance across sexes and geographic regions. This innovation holds promise for improving CAD diagnosis by offering a noninvasive point-of-care solution. The developed model can be seamlessly integrated into an all-in-one technology, like the CorVista Capture Device, which is equipped with PPG and OVG sensors capable of collecting and transmitting signals to a cloud-based analyzer through Wi-Fi or data networks, thereby facilitating the prediction of CAD at the point of care. To advance this goal, further steps for this study include validation on a larger clinical dataset to confirm the performance and generalizability of the model before considering implementation in clinical practice.

## Figures and Tables

**Table 1:** Function of each dataset in the CAD algorithm development.

Dataset	Total Subjects	Function	
		Feature Selection	Training and Internal Validation *
CADLAD	624	N = 208 CAD− N = 208 CAD+	N = 416 CAD+ **
IDENTIFY Group 2	225	None	N = 225 CAD+
IDENTIFY Group 4	513	None	N = 513 CAD−
Total	1362	416	1154

* Training and internal validation is the intended use dataset; ** Including the N = 208 CAD+ from feature selection.

**Table 2:** Demographics and disease.

Characteristic *	CAD− IDENTIFY G4	CAD+ CADLAD and IDENTIFY G2	*p*-Value
Number of Subjects	513	641	
Age			
Mean ± STD	55 ± 12.0	64.9 ± 9.6	<0.05
Age ≥ 65	23.0%	56.3%	<0.05
Female	64.5%	26.7%	<0.05
BMI			
Mean ± STD	31.3 ± 6.6	30.9 ± 6.2	0.316
BMI ≥ 30	52.2%	54.3%	0.526
Hypertension	60.0%	78.6%	<0.05
Diabetes	15.8%	34.5%	<0.05
Hyperlipidemia	52.6%	76.6%	<0.05
Degree of CAD **			
CADRADS 0	43.5%		
CADRADS 1	26.7%		
CADRADS 2	29.6%		
Single-vessel		48.8%	
Multi-vessel		51.2%	

* Continuous characteristics are represented as mean ± standard deviation, and categorical characteristics are represented as a percentage. ** Definition for the CADRADS is provided in the table given in Supplementary Section S2.

**Table 3:** (**a**) CAD Algorithm performance statistics; (**b**) CAD Algorithm 2 × 2 table.

(**a**)				
**Group**	**ROC-AUC**	**Cut Point**	**Sensitivity**	**Specificity**
Female	0.87	0.482	90%	61%
Male	0.81	0.361	90%	54%
Both Sexes	0.85	n/a	90%	59%
CAD+ Subjects used for FA and ML	n/a	n/a	88%	n/a
(**b**)				
**CAD Test Result**	**Actual CAD Status**			
	**CAD+**	**CAD−**		
Test-Positive	574	212		
Test-Negative	67	300		
			Odds Ratio = 12.12	

**Table 4:** Multivariate logistic regression to assess for bias in the CAD algorithm performance.

Predictor	Odds Ratio (95% CI)	*p*-Value
Test-Positive CAD Score	7.50 (5.29, 10.64)	<0.05
Male	5.81 (4.22, 8.00)	<0.05
Age (≥65)	3.20 (2.31, 4.44)	<0.05
Diabetes	2.37 (1.64, 3.43)	<0.05
Hyperlipidemia	1.99 (1.43, 2.77)	<0.05
Hypertension	1.21 (0.86, 1.71)	0.277

## Data Availability

Relevant deidentified subsets of the dataset may be shared with academic investigators on a case-by-case basis.

## Supplementary Materials

The following supporting information can be downloaded at https://www.mdpi.com/article/10.3390/diagnostics14070719/s1, Section S1: Study Inclusion/Exclusion Criteria; Section S2: CADRADS Definition; Section S3: Additional Metrics for the Model Performance; Section S4: Model Subgroup Performance; Section S5: Features. References [34,35] are cited in the Supplementary Materials.
