# Supplementary material for: Development of a Non-Invasive Machine-Learned Point-of-Care Rule-Out Test for Coronary Artery Disease
Source: Diagnostics (Basel). 2024 Mar 28;14(7):719. doi: 10.3390/diagnostics14070719 (PMC11012183; doi:10.3390/diagnostics14070719)
Supplement: Supplementary file 1 [file diagnostics-14-00719-s001.zip › diagnostics-2929911-supplementary.pdf]

Supplement Section S1 – Study Inclusion / Exclusion Criteria

Inclusion	Exclusion
1. ≥ 18 years of age<br>2. Cardiovascular symptoms<br>3. Scheduled to undergo cardiac catheterization with coronary angiography (Group 2) or Computed Tomography Angiography (Group 4)<br>4. Ability to understand the requirements of the study and to provide written informed consent	1. Prior documented history of myocardial infarction (MI)<br>2. Suspected acute myocardial infarction (AMI) at current presentation<br>3. Prior coronary artery bypass grafting (CABG)<br>4. Prior heart valve replacement<br>5. Previous sustained or paroxysmal atrial or ventricular arrythmia<br>6. Infiltrative myocardial disease (amyloid, sarcoid, right ventricular dysplasia)<br>7. Presence of cardiac implantable electronic device (CIED), including implantable cardioverter defibrillator (ICD), pacemaker (PM), implantable loop recorders and other monitors<br>8. Implantable Neuro-stimulators<br>9. Congenital Heart Disease<br>10. Pregnant or breast feedingCurrently taking any Type IA, IC or III antiarrhythmics<br>11. Any history of amiodarone useClinically significant chest deformity (e.g., pectus excavatum or pectus carinatum)<br>12. Breast implants<br>13. Neuromuscular disease if the condition results in tremor or muscle fasciculations

Supplement Section S2 – CADRADS Definition

CADRADS categories and management for patients with stable chest pain (symptomatic)

Category	Degree of Maximal Coronary Stenosis	Interpretation	Further Cardiac Investigation	Management considerations
**CAD-RADS 0**	0%	Absence of CAD<br>(No plaque or stenosis)	None	Reassurance. Consider non-atherosclerotic causes of symptoms
**CAD-RADS 1**	1-24%	Minimal non-obstructive stenosis<br>(Minimal plaque with no stenosis)	None	Consider non-atherosclerotic causes of symptoms<br>P1: Consider risk factor modification and preventive pharmacotherapy<br>P2: Risk factor modification and preventive pharmacotherapy<br>P3 or P4: Aggressive risk factor modification and preventive pharmacotherapy
**CAD-RADS 2**	25-49%	Mild non-obstructive stenosis	None	Consider non-atherosclerotic causes of symptoms<br>P1 or P2: Risk factor modification and preventive pharmacotherapy<br>P3 or P4: Aggressive risk factor modification and preventive pharmacotherapy

Supplement Section S3 – Additional Metrics for the Model Performance

	BOTH SEXES	MALES	FEMALES
**MCC***	0.51	0.47	0.48
**F1 SCORE**	0.80	0.86	0.67
**AUC-PR****	0.88	0.91	0.79

* Matthews Correlation Coefficient (MCC)

** Area under the precision-recall curve (AUC-PR)

Supplement Section S4 – Model Subgroup Performance

Subgroup	AUC	Sensitivity	Specificity
Female	0.87	90%	61%
Male	0.81	90%	54%
Age < 65	0.84	85%	66%
Age ≥ 65	0.81	93%	35%
BMI < 30	0.85	88%	59%
BMI ≥ 30	0.85	91%	58%
Non-Diabetic	0.86	90%	62%
Diabetic	0.81	89%	42%
Non-Hypertensive	0.86	84%	71%
Hypertensive	0.83	91%	50%
Non-Hyperlipidemic	0.87	90%	64%
Hyperlipidemic	0.84	89%	54%

Arterial Compliance Features: This category of features captures arterial compliance (i.e., increasing stiffness) using the velocity plethysmogram (VPG) and acceleration plethysmogram (APG). VPG and APG signals are generated by taking the first and second derivatives of the PPG signal. It has been widely reported that the shapes of the PPG, VPG, and PPG waveforms are associated with arterial compliances [1]. Herein, PPG and its derivatives are characterized in the time domain and features such as amplitude, duration, and intervals are extracted. Phase space, a three-dimensional representation of PPG-VPG-APG, is constructed and features capturing the interactions between the PPG modalities in the three-dimensional and two-dimensional space are extracted (e.g. length of the loop, 3D vector angles, 3D vector magnitude, curvature, etc.). An example of two-dimensional phase space VPG-PPG used to extract arterial compliance features is shown in Figure S1.

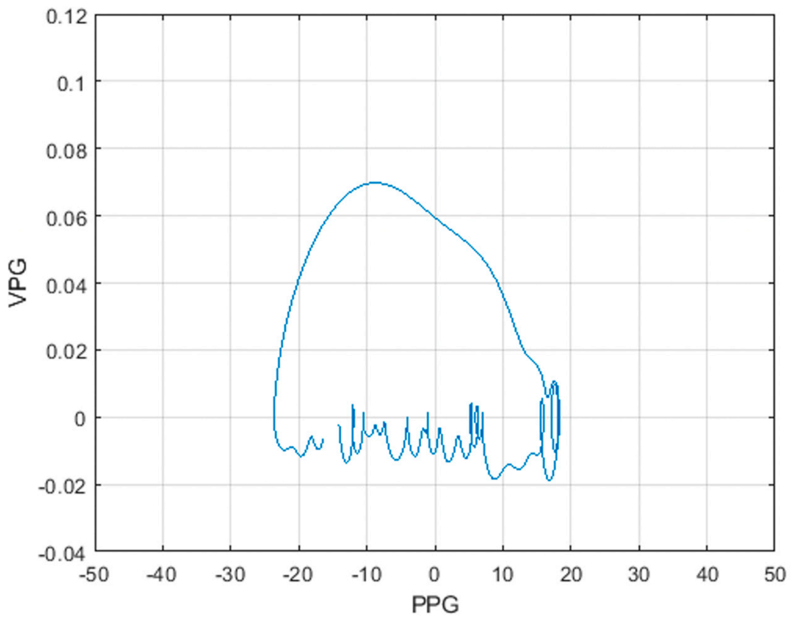

(a) CAD- Example

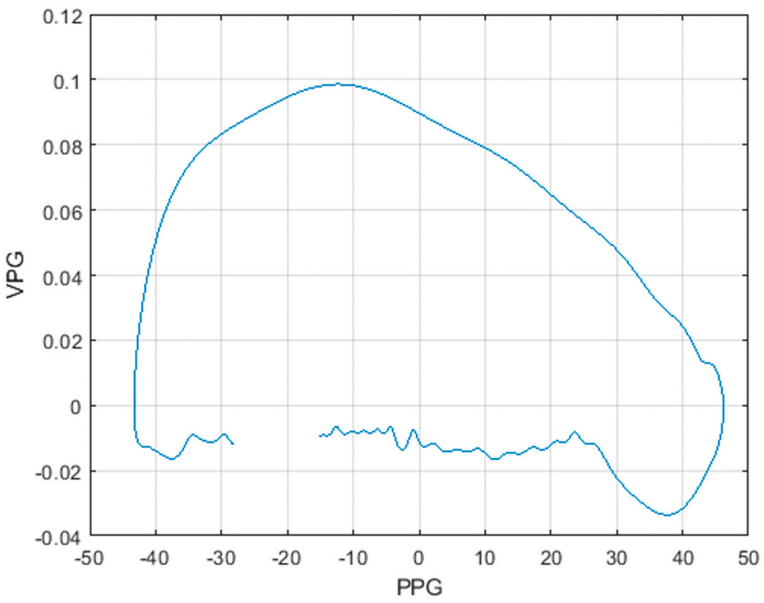

(b) CAD+ Example

Figure S1: (a) Example of a CAD- subject exhibiting a smaller VPG-PPG 2D loop feature value (e.g. area, perimeter, maximal 2D vector magnitude) versus (b) and a CAD+ subject exhibiting a large VPG-PPG 2D loop feature value.

Conduction Features: These features measure characteristics of myocardial conduction pathway and variations in that pathway using the OVG signal. Studies showed that Heart rate variability and microvolt T wave alternans changes have predictive power for the detection of CAD and other cardiac diseases [2], [3]. Herein, conduction variability is computed using the template matching technique, which is based on generation of a representative template cardiac cycle using all the cycles acquired in the signal, and then assessing deviations of each individual cycles to that template. All the cardiac cycles are stacked on the cardiac template using the R-peak as the fiducial point and then assessing deviations of each individual cycle to that template for each of the three cardiac channels, called residue waveform. In total, 31 features are engineered using this method by looking at the distribution of the residue from the template beat (i.e. mean, standard-deviation, skew, etc.) and the three-dimensional shape of point-cloud the residue (i.e. volume, surface area, etc.). Similar to the concept of heart rate variability, non-diseased subjects will exhibit normal variation in the cardiac cycle. In contrast, diseased subjects will present with either very little or substantial variation. Examples of template signal and the stacked cardiac cycle used for the creation of the residue waveforms used for the calculation of the conduction features ~~the cycle variability feature~~ are presented in Figure S2.

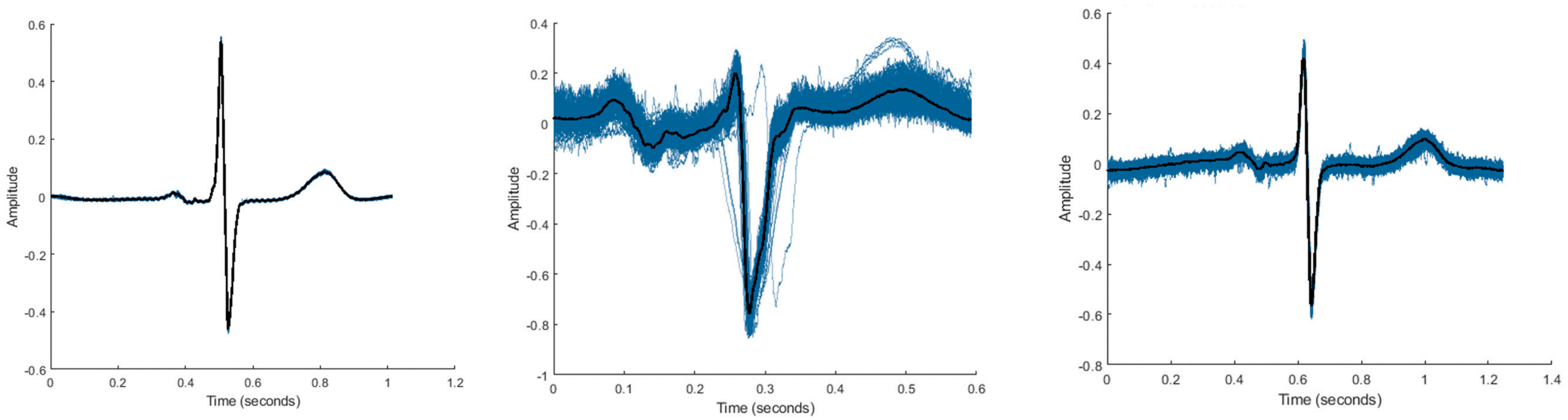

(a) CAD+ *(b) CAD+* *(c) CAD-*

Figure S2: (a) Example of a CAD+ subject exhibiting low conduction cycle variability, (b) CAD+ subject exhibiting high conduction cycle variability, and (c) a CAD- subject exhibiting normal conduction cycle variation.

Perfusion Features: These features capture the ~~shape of~~ information embedded in the PPG waveform itself, as well as the relationship between the infrared and red PPG signals; Continuous wavelet transform (CWT) is employed to transform the PPG, VPG, and APG signals for the time domain to time-frequency domain and generate CWT scalogram. The PPG modalities are delineated using the fiducial point from the OVG signal matching the corresponding to the cardiac cycle. the obtained scalograms are divided into regions with different energy levels (high energy regions are shown by dash-line in Figure S3) using image processing and statistical techniques. Examples of these regions are presented in Figure S3, where the PPG waveform is overlaid on the CWT scalogram for a case with no secondary region detected (Figure S3.a) and a case with two regions of high energy (Figure S3.b). Image-based features (i.e. area, time centroid, frequency centroid, maximum energy, time span, frequency span, number of regions, etc.) are extracted from these regions within the scalograms. For example, wavelet time-frequency analysis is used to detect and characterize each high-power time-frequency region in the PPG, and specifically, detect a secondary peak in the power spectrum typically associated with a secondary peak in PPG waveform itself (i.e., dicrotic notch). Examples of this feature are presented in Figure S3. The distribution of the feature in the Intended Use Dataset is also presented, since it is of particular interest due to the formation of a bimodal distribution, with CAD+ subjects being more frequent in the mode with the absence of the secondary peak in the spectrum.

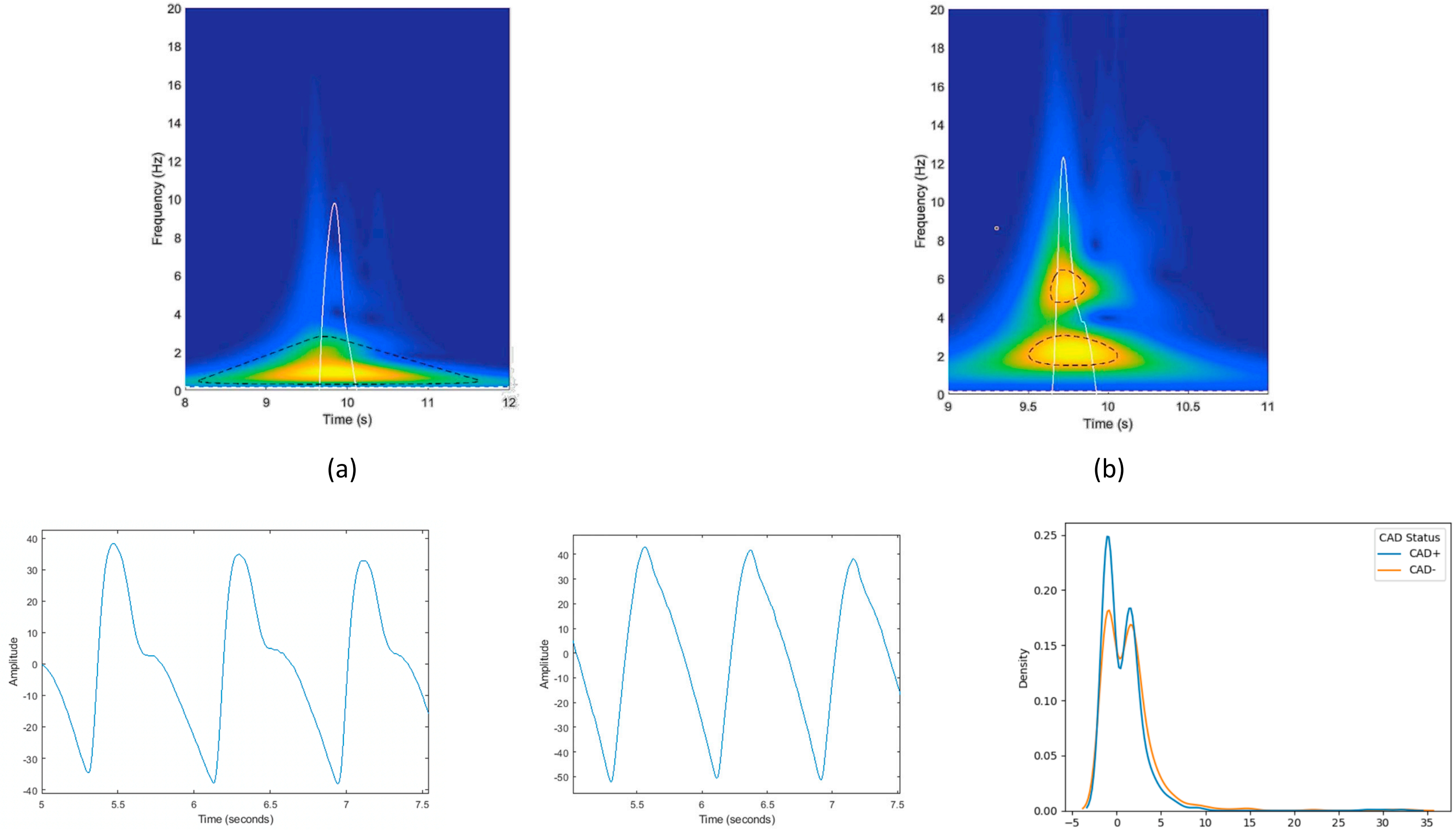

(c) CAD- *(d) CAD+* *(e) Distribution*

Figure S3: (a) Example of a PPG scalogram with single detected high energy region, (b) example of a PPG scalogram with two detected high energy regions, (c) example of the perfusion feature corresponding the energy distribution in high power and low power regions for CAD- subject exhibiting, (d) example of the perfusion feature corresponding the energy distribution in high power and low power regions for CAD+ subject, and (e) the distribution of the feature by CAD status.

Repolarization Features: These features measure the properties of myocardial repolarization. Similar to the PPG perfusion feature, computation of the repolarization features is based on the CWT scalograms for the segmented T-wave and the detection of the high-power regions for it. The T-waves are delineated and segmented using a discrete wavelet-based delineator. An Example of a wavelet scalogram for the T-wave is shown in Figure S4. Similar to perfusion features, image-based features are extracted from the generated scalograms. For example, in Figure S4, it can be seen that the duration of the high energy region tends to exhibit larger values for a CAD+ subjects, corresponding to longer repolarization, which is a characteristic indicative of repolarization deficits.

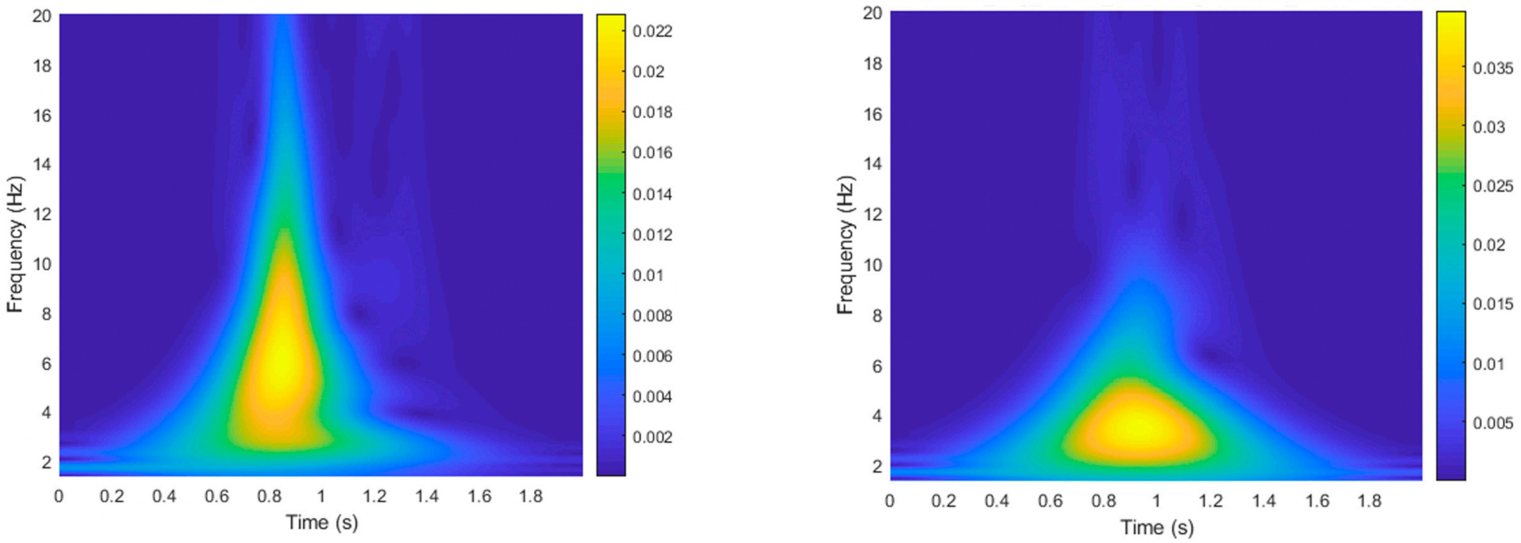

(a) CAD- *(b) CAD+*

Figure S4: Example of repolarization feature (a) a CAD- subject exhibiting shorter time span for the high energy region (b), a CAD+ subject exhibiting longer time span for the high energy region.

Perfusion Response to Cardiac Contraction: These features analyze the relationship of the PPG signal to the OVG signal – dynamics of the interplay between blood flow and myocardial conduction characteristics could indicate differential cardiac function, reflecting underlying pathophysiology. Detailed information on calculating this set of features is presented elsewhere [4]. In one example,

shown in Figure S5, the cross-correlation between the OVG and PPG signal is calculating, displaying a well-defined bimodal distribution, with CAD+ subjects tending to have lower lag values and CAD- with higher lag values.

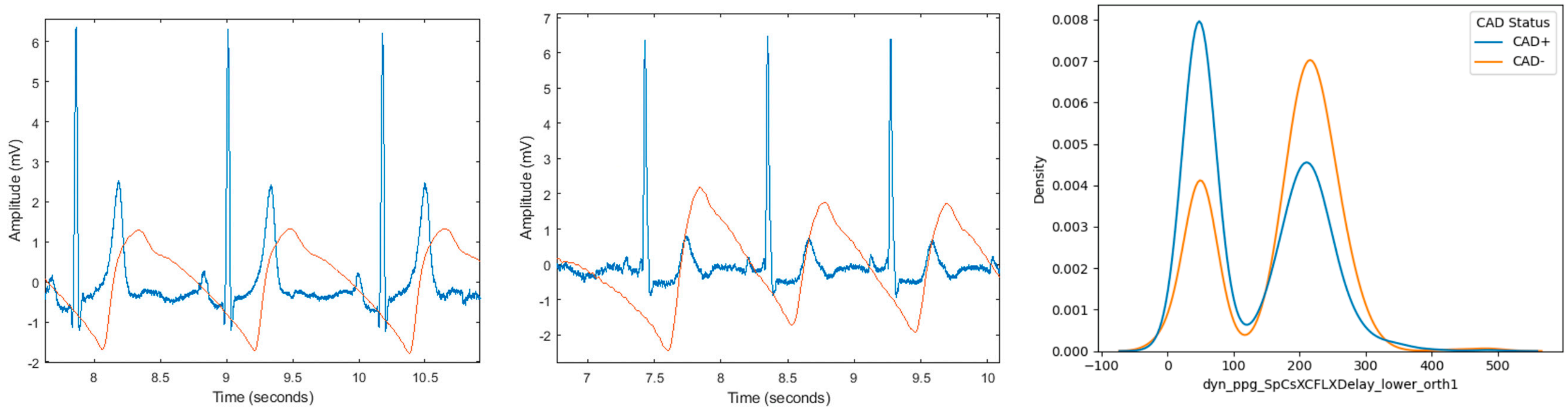

(a) CAD- Example *(b) CAD+ Example* *Distribution (c)*

Figure S5: (a) Example of a CAD-subject exhibiting negative perfusion response to cardiac contraction (PRCC) feature value, (b) a CAD+ subject exhibiting a positive PRCC feature value, and (c) the distribution of the feature by CAD status (c).

Atrial Structure: These features capture elements of atrial structure in the time domain, such as amplitude, duration, peak terminal time, number of peaks, shape of the wave, etc. For example, Figure S6 presents the complexity of atrial depolarization through the presence of secondary waveforms in the P-wave for the CAD+ patient. Features such as peak distances (distance from the first peak to the second peak), amplitude ratio, and relative wave duration are extracted to quantify such patterns in the P-wave.

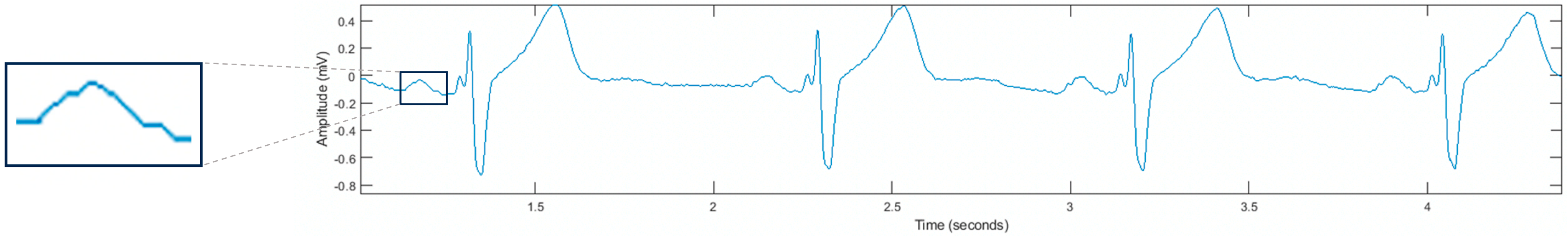

(a) CAD- Example (a) – first p-wave (left) & first 4 cardiac cyles (right)

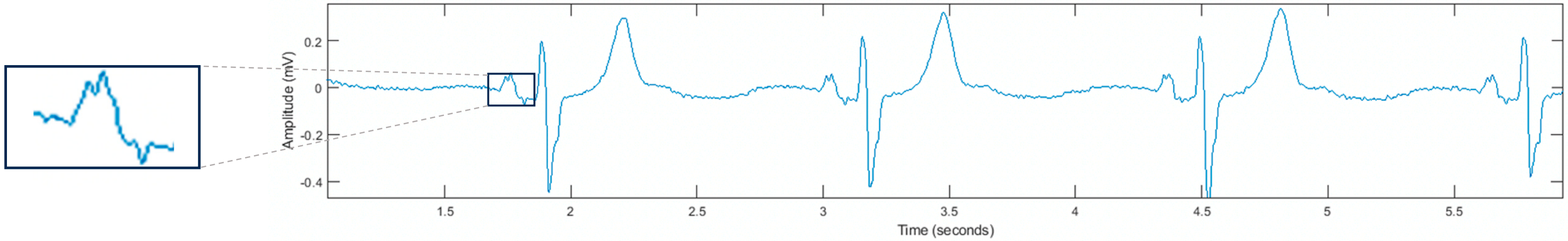

(b) CAD+ Example (a) – first p-wave (left) & first 4 cardiac cyles (right)

Figure S6: (a) Example of a CAD subject exhibiting a negative atrial structure feature value and (b) a CAD+ subject exhibiting a positive atrial structure feature value.

References

[1] M. Elgendi, “On the analysis of fingertip photoplethysmogram signals,” *Curr. Cardiol. Rev.*, vol. 8, no. 1, pp. 14–25, 2012.

[2] D. Bansal, M. Khan, and A. K. Salhan, “A Review of Measurement and Analysis of Heart Rate Variability,” in *2009 International Conference on Computer and Automation Engineering*, 2009, pp. 243–246, doi: 10.1109/ICCAE.2009.70.

[3] P. A. Călburean *et al.*, “Heart rate variability and microvolt T wave alternans changes during ajmaline test may predict prognosis in Brugada syndrome,” *Clin. Auton. Res.*, vol. 33, no. 1, pp. 51–62, Feb. 2023, doi: 10.1007/s10286-023-00922-4.

[4] F. Fathieh *et al.*, “Predicting cardiac disease from interactions of simultaneously-acquired hemodynamic and cardiac signals,” *Comput. Methods Programs Biomed.*, vol. 202, p. 105970, 2021.
